# Integrated Monitoring of Water Quality, Metal Ions, and Antibiotic Residues, with Isolation and Optimization of Enrofloxacin-Degrading Bacteria in American Shad (*Alosa sapidissima*) Aquaculture Systems

**DOI:** 10.3390/jox15060174

**Published:** 2025-10-22

**Authors:** Yao Zheng, Jiajia Li, Ampeire Yona, Xiaofei Wang, Xue Li, Julin Yuan, Gangchun Xu

**Affiliations:** 1 Wuxi Fisheries College, Nanjing Agricultural University, Wuxi 214081, China; 2 Key Laboratory of Freshwater Fisheries and Germplasm Resources Utilization, Ministry of Agriculture and Rural Affairs, Freshwater Fisheries Research Center (FFRC), Chinese Academy of Fisheries Sciences (CAFS), Wuxi 214081, China; 3 Key Laboratory of Healthy Freshwater Aquaculture, Ministry of Agriculture and Rural Affairs, Key Laboratory of Fish Health and Nutrition of Zhejiang Province, Zhejiang Institute of Freshwater Fisheries, Huzhou 313001, China

**Keywords:** antibiotic degradation, healthy culture, pond sediment, season change, water source

## Abstract

This study investigated water quality, metal ion concentrations, and antibiotic residues specifically enrofloxacin (ENR) and its metabolite ciprofloxacin (CIP), across six American shad (*Alosa sapidissima*) aquaculture sites over a one-year period. Water and sediment samples were analyzed to determine contamination levels, and ENR-degrading bacteria were isolated from the culture environment to explore their potential use in bioremediation. Findings showed that NH_3_-N and total suspended solids (TSS) exceeded recommended standards at all sampling sites. Elevated levels of Li, Na (except S1), Fe, Ni (except S2 and S4), Sr, and Cu were found at site S3. Site S5 recorded the highest concentrations of Al, As, and Pb, while Cd was most abundant at S6. In sediments, S5 showed higher levels of Mg, K (except S3), Ca, Cr, Mn, Fe, Ni, As, Pb, Cu, and Zn (except S3). ENR and CIP were detected in all water and sediment samples, with a 100% detection rate. The highest ENR (16.68–3215.95 mg·kg^−1^) and CIP (3.90–459.60 mg·kg^−1^) concentrations in water occurred at site S6, following a seasonal pattern of autumn > winter > summer > spring. In sediments, the maximum ENR (41.43–133.67 mg·kg^−1^) and CIP (12.36–23.71 mg·kg^−1^) levels were observed in spring. Two ENR-degrading bacterial strains were successfully isolated and identified as *Enterococcus* and *Bacillus*. Optimal degradation was achieved at 30 °C, pH 8.0, 6% inoculum, and 3000 Lux, resulting in a 64.2% reduction in ENR after 72 h. Under slightly different conditions (25 °C, pH 10), degradation reached 58.5%. This study provides an efficient strain resource for the bioremediation of ENR pollution in the aquaculture water of American shad.

## 1. Introduction

The American shad (*Alosa sapidissima*), originally introduced from the United States [1], has become one of the most valuable aquaculture species in China. In premium restaurants, it can sell for as much as 80–100 USD per kilogram, making it a luxury product in the domestic market. This high economic value has spurred a wide range of research, including studies on reproductive biology [2,3], nutritional composition, and even applications such as collagen extraction for biomaterials [4,5,6]. Despite these advances, farming American shad remains difficult. Outbreaks of bacterial and parasitic diseases are frequent, and the species is highly sensitive to changes in its environment [5,6]. These challenges are particularly acute in recirculating aquaculture systems (RAS) and reservoir cage farming, where shad are commonly raised.

Water quality plays a central role in the success of American shad farming. Elevated concentrations of toxic metals, such as cadmium (Cd), can reduce survival and growth while also threatening product safety [7]. Heavy metals have been found in water and sediment samples in lakes of Europe [8]. In intensive aquaculture systems, hydrodynamics further complicate management by altering the pharmacokinetics of administered drugs. For example, flowing water has been shown to accelerate the clearance of norfloxacin from fish tissues, while simultaneously promoting its accumulation [9]. To combat bacterial infections, antibiotics such as enrofloxacin (ENR) are widely applied in Chinese aquaculture [10]. However, frequent and often excessive use has resulted in the persistence of ENR in surrounding waters, sediments, and even fish tissues. Long-term exposure has been associated with oxidative stress, metabolic disruption, and inflammation in cultured species, including American shad [10,11].

Monitoring studies across China have confirmed the widespread occurrence of ENR and its major metabolite, ciprofloxacin (CIP), in aquaculture environments. In Lake Honghu, for instance, ENR was detected in water, sediments, and fish tissues throughout the year, sometimes reaching several micrograms per kilogram [12,13]. Higher levels have been reported in fish ponds, compared to shrimp or crab ponds, in Lake Taihu [14]. In Guangdong, ENR residues in pond water and sediments reached 21.3 ng·L^−1^ and 446 μg·kg^−1^, respectively [15], while in Laizhou Bay marine farms, residues were also found in both water and sediment samples [16]. These compounds not only persist in the environment but also accumulate in fish tissues, like in rainbow trout of some European countries [17] raising concerns about bioaccumulation, food safety, and the spread of antibiotic resistance genes (ARGs) [18,19].

The ecotoxicological risks of ENR are significant. The predicted no-effect concentration for algae is only 4.9 μg·L^−1^ [20], and in tilapia, ENR displays a long elimination half-life (21.7 h) and low clearance rate (0.09 L·h^−1^·kg^−1^), suggesting strong persistence in aquatic organisms [21]. For American shad specifically, ENR exposure has been linked to gut microbiota disruption, oxidative stress, and altered metabolic pathways that influence growth and body weight [10,11].

Environmental conditions strongly shape the fate of ENR. Factors such as light intensity, temperature, pH, salinity, and initial drug concentration all influence its degradation. Advanced physicochemical treatments, including photocatalysis with Bi_2_WO_6_ or TiO_2_ [22,23] and electro-Fenton oxidation [24], have shown high efficiency under controlled laboratory conditions. Yet these methods remain costly and difficult to apply in large-scale aquaculture. Biodegradation by naturally occurring bacteria offers a more practical and sustainable alternative. Strains such as *Providencia*, *Enterobacter*, *Alcaligenes*, *Bacillus subtilis*, and *Pseudomonas aeruginosa* have been reported to degrade ENR [25]. These processes are often driven by resistant bacteria, with other microbial communities contributing to the breakdown pathways. However, ENR can also suppress beneficial groups, such as ammonia-oxidizing bacteria, and interactions with metal ions may further complicate degradation dynamics [26].

Given these challenges, there is a clear need for a dual approach: systematic monitoring of water quality, heavy metal, metal ions, and antibiotic residues in American shad aquaculture, coupled with the isolation and optimization of indigenous bacterial strains capable of degrading ENR. The present study addresses these issues by (i) assessing pollution levels in multiple shad culture environments, with a focus on ENR and CIP, and (ii) screening and characterizing ENR-degrading bacteria, while identifying the environmental conditions that maximize their degradation efficiency.

## 2. Materials and Methods

### 2.1. Sample Collection and Experimental Setup

**Figure 1:**
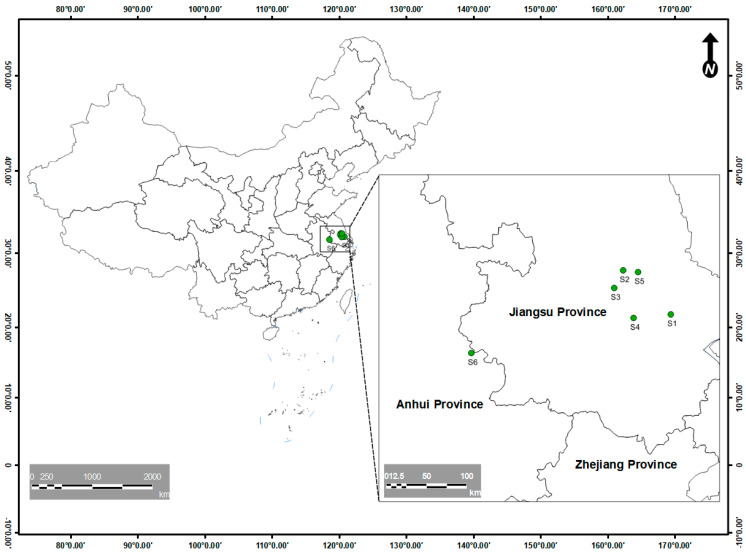
Location of the sampling points.

Water (including source and pond), sediment samples (*n* = 3) were picked from six American shad culture places, including Wuxi Long Chao Ecological Agricultural Technology Co., Ltd. (culture density 4.5 individuals·(m^2^)^−1^ named as S1, 120°44′ E, 31°66′ N, Wuxi, China, Figure 1), the greenhouse pond of Jiangyin Shengyang Sanxian Breeding Co., Ltd. (S2, culture density 4 individuals·(m^2^)^−1^, 120°12′ E, 31°91′ N, Wuxi, China), Changzhou Noah ark Agricultural Technology Co., Ltd. (S3, culture density 6 individuals·(m^2^)^−1^, 120°06′ E, 31°81′ N, Changzhou, China), Changzhou Tanshi special Aquaculture Co., Ltd. (S4, culture density 7.5 individuals·(m^2^)^−1^, 119°79′ E, 31°64′ N, Changzhou, China), Yangzhong Jiang Zhiyuan, Food Industry Co., Ltd. (S5, culture density 4 individuals·(m^2^)^−1^, 119°82′ E, 32°30′ N, Yangzhong, China), Anhui Changjiangyuge Fishery Co., Ltd. (S6, culture density 7–8 individuals·(m^2^)^−1^, 118°30′ E, 31°44′ N, Wuhu, China). The samples were picked from 10th January 2022 to 10th January 2023 every month. The water samples were picked in acid-washed HDPE 5-liter transparent containers (Nalgene®, Sigma-Aldrich, Nanjing, China). Sediments weighing approximately 1 kg were collected from the top 15 cm of each sampling point, placed in plastic bags tightly zipped, and transported to the lab in a cooler.

### 2.2. Water Quality and Metal Ion Determination for Each Sample Point

Water samples were analysed in the laboratory for water quality, and the parameters were selected based on potential pollutants from aquaculture. The parameters included total nitrogen (TN), total phosphate (TP), ammonia nitrogen (NH_3_-N), chemical oxygen demand (COD_Mn_), total suspended solids (TSS), and pH. Heavy metal and metal ion determination was performed by ICP-MS. Analysis for copper ions was carried out by means of graphite furnace atomic absorption spectrometry [27,28]. For sediment exchangeable Cu, 25 mL of 1 M NH_4_NO_3_ was used to extract 1.0 g of oven-dried sediment (105 °C for 12 h). In the lab, water samples were saved to pH forms utilizing air-segmented continuous autoflow analysers after being 0.45 µm filtered. Before extraction and analysis, sediment samples were air-dried and put through a #10 sieve.

### 2.3. Enrofloxacin and Ciprofloxacin Determination

The water samples were first filtered through 0.45 μm glass fiber filters (Pall Corp., Pensacola, FL, USA), and 500 mL of the filtrate was collected. To this, 0.2 g of Na_2_EDTA was added, and the mixture was shaken thoroughly. The pH was then adjusted to approximately 5.0 using formic acid. The solution was enriched using an Oasis HLB column (200 mg, 6 mL; Waters Corporation, Sarasota, FL, USA). The column was conditioned with 10 mL of methanol followed by 10 mL of ultrapure water before extracting the water sample. Target analytes were eluted with 8 mL of methanol. The collected eluate was concentrated to near dryness using a rotary evaporator at 50 °C and then reconstituted to 1 mL with 20% methanol in acidified water (0.1% formic acid) after filtration through a 0.22 μm membrane. Sediment samples (≥50 g) were first passed through a 2 mm nylon sieve, air-dried, and then ground until the particles passed through a 0.15 mm nylon sieve.

A liquid chromatography tandem mass spectrometry (LC-MS/MS) method was used for the simultaneous quantification of the concentrations of ENR [29]. Approximately 2.00 ± 0.02 g of each sample was accurately weighed into a 50 mL centrifuge tube. Ten milliliters of extraction solvent (0.1% formic acid in acetonitrile) was added, and the mixture was vortexed at 2000 r·min^−1^ for 15 min, followed by centrifugation at 12,000 r·min^−1^ for 5 min. The solid-phase extraction column was activated and equilibrated sequentially with 5 mL of methanol and 5 mL of water. Two milliliters of the resulting supernatant was passed through the column at a controlled flow rate, and the analytes were eluted twice using formic acid–methanol solution (3 mL and 2 mL) into a 200 mL round-bottom flask. The eluate was evaporated to dryness in a 45 °C water bath using a rotary evaporator under a vacuum of 0.07 MPa. The residue was reconstituted in 1 mL of the initial mobile phase and filtered through a 0.22 μm PTFE organic-phase membrane filter.

Chromatographic separation was performed on an Agilent ZORBAX SB-C18 column (150 × 4.6 mm, 5 µm, Santa Clara, CA, USA). The mobile phase consisted of acetonitrile and 0.01 mol·L^−1^ tetrabutylammonium bromide solution (pH adjusted to 2.8 with phosphoric acid) at a ratio of 10:90 (*v*/*v*). The mobile phase was filtered, degassed, and equilibrated for 25 min before use. The column temperature was maintained at 30 °C, with a flow rate of 1.0 mL·min^−1^. Detection was carried out using a fluorescence detector with an excitation wavelength of 280 nm and an emission wavelength of 450 nm. The injection volume was 10 µL.

### 2.4. Screening of ENR Degradation Bacteria

Standard solutions of enrofloxacin (ENR) and ciprofloxacin (CIP) were prepared by accurately weighing 0.01 g of each compound, dissolving them in distilled water, and diluting to 200 mL in brown volumetric flasks. From these stock solutions, standard working solutions with concentrations of 250, 100, 50, 5, and 1 μg·L^−1^ were prepared. Calibration curves were established by plotting concentration (μg·L^−1^) against peak area, yielding the regression equations *Y* = 1014.2*X* − 1275.6 (*R*^2^ = 0.9998) for ENR and *Y* = 503.6*X* − 625.4 (*R*^2^ = 0.9999) for CIP. These equations were used to determine ENR concentrations in the collected water and sediment samples. Samples with the lowest ENR content were adjusted to a final concentration of 200 μg·L^−1^ for further degradation studies.

For bacterial enrichment, 10 mL of pond water was added to a 250 mL conical flask containing 90 mL of sterile aerobic denitrification medium and incubated at 30 °C on a rotary shaker at 150 r·min^−1^ for two days. Then, 10 mL of the culture was transferred to 90 mL of fresh medium and incubated for three additional days under the same conditions. This subculturing step was repeated three times to enhance the enrichment of ENR-degrading bacteria. The enriched cultures were serially diluted in phosphate buffer solution (10^2^–10^7^), and 0.1 mL from each dilution was spread on solid separation media. Plates were inverted and incubated at 30 °C until distinct single colonies appeared, from which purified strains were obtained.

Each purified strain was inoculated into 90 mL of Luria–Bertani (LB) broth containing 200 μg·L^−1^ ENR and incubated at 30 °C with shaking at 150 rpm for 48 h. After incubation, nitrate nitrogen (NO_3_^−^–N) and total nitrogen (TN) concentrations in the medium were determined spectrophotometrically, and the strain showing the highest denitrification activity was selected for further analysis.

Genomic DNA from the selected strains was extracted using a commercial bacterial DNA extraction kit. The 16S rRNA gene was amplified by PCR using universal primers 27F (5′-AGGTTTGATCCTGCTCAG-3′) and 1492R (5′-GGTTTACCTTGTTACGACTT-3′). The amplification protocol consisted of an initial denaturation at 95 °C for 3 min, followed by 35 cycles of denaturation at 95 °C for 30 s, annealing at 60 °C for 30 s, and extension at 72 °C for 30 s, with a final extension at 72 °C for 5 min. TIANGEN polymerase was used for all reactions. PCR products were verified by agarose gel electrophoresis and sent to Shanghai Bioengineering Co., Ltd., Shanghai, China for sequencing. The resulting sequences were compared against the NCBI database using BLAST, and phylogenetic relationships were analyzed using MEGA12 software.

### 2.5. Optimization Conditions for ENR Degradation

Temperature gradients were set at 15, 20, 25, and 30 °C with an inoculum volume of 2% and an initial pH of 7.0. To examine the influence of pH, initial values of 4.0, 6.0, 7.0, 8.0, and 10.0 were tested while maintaining a 2% inoculum and a temperature of 30 °C. The effect of inoculum size was evaluated at 2%, 4%, 6%, and 8%, with the initial pH maintained at 7.0 and temperature at 30 °C. Light intensity was tested at 0, 3000, and 6000 Lux under the same inoculum volume (2%), pH (7.0), and temperature (30 °C). Cultures containing ENR concentrations ranging from 5 to 200 μg·L^−1^ were incubated for 96 h under these conditions.

Each treatment was performed with three biological replicates, and an uninoculated medium containing 200 μg·L^−1^ ENR served as the blank control. These experiments were conducted to determine the optimal temperature, pH, inoculation volume, and illumination intensity for bacterial degradation of ENR. Once the optimal conditions were identified, a combined optimization experiment was carried out in triplicate, with each test repeated three technical times for accuracy.

The degradation efficiency (%) was calculated using the formula: Degradation rate (%) = (*y*0 − *yt*)/*y*0 × 100, where $y_{t}$ represents the antibiotic concentration at time t, and $y_{0}$ is the initial concentration.

### 2.6. Data Statistical Analysis

All experimental data were processed using SPSS 26.0 software and presented as mean ± standard deviation (SD). To statistical testing, data that did not conform to normal distribution or homogeneity of variance were log_2_-transformed to meet the assumptions of parametric analysis. Redundancy analysis (RDA) was used for determining variability of the water quality state parameters and to identify the key quality factors that cause variability [30].

One-way analysis of variance (ANOVA) was employed to assess differences among treatment groups. When significant variation was detected (*p* < 0.05), Tukey–Kramer post hoc tests were conducted to determine specific group differences. A significance level of *p* < 0.05 was considered statistically meaningful throughout all comparisons.

## 3. Results

### 3.1. Water Quality, Heavy Metals and Metal Ions

The concentrations of total nitrogen (TN) and total phosphorus (TP) in the water samples ranged from 1.22 to 13.30 mg·L^−1^ and 0.05 to 0.80 mg·L^−1^, respectively. Among these, site S4 exceeded the standard limit for TN (6 mg·L^−1^). Ammonia nitrogen (NH_3_-N) and total suspended solids (TSS) were above the acceptable limits at all sampling points, whereas permanganate index (CODₘₙ) and pH values remained within the standard range (Table 1). Overall, the water quality at site S3 appeared to be better than that of the other sites.

Metal analysis showed that the concentrations of Li (8.71–136.51 μg·L^−1^), Na (2556.60–17 753.26 μg·L^−1^, except at S1), Fe (ND–1.07 μg·L^−1^), Ni (0.38–2.86 μg·L^−1^, except at S2 and S4), Sr (171.20–562.85 μg·L^−1^), and Cu (1.25–7.77 μg·L^−1^) were higher at S3 than at the other sites (Table 2). In contrast, S4 exhibited elevated levels of Mg (1419.67–3740.67 μg·L^−1^), Ca (2261.61–7150.73 μg·L^−1^, except at S2), Mn (ND–5.00 μg·L^−1^), Co (0.06–0.19 μg·L^−1^), and Ba (3.65–18.23 μg·L^−1^, except at S6) (Table 3). The highest concentrations of Al (0.32–0.83 μg·L^−1^), As (1.07–9.82 μg·L^−1^), and Pb (ND–19.98 μg·L^−1^) were observed at S5, while K (236.39–684.50 μg·L^−1^) peaked at S1 and Cd (ND–0.05 μg·L^−1^) at S6. Selenium (0.98–1.38 μg·L^−1^) and Zn (0.06–0.07 μg·L^−1^) showed little variation among the sampling sites.

In sediment samples, with the exception of S4, the concentrations of Mg (65.49–294.87 mg·kg^−1^), K (39.56–69.29 mg·kg^−1^, except S3), Ca (168.85–693.51 mg·kg^−1^), Cr (7.80–12.72 mg·kg^−1^), Mn (11.97–29.94 mg·kg^−1^), Fe (450.47–762.26 mg·kg^−1^), Ni (5.16–10.18 mg·kg^−1^), As (1.13–6.27 mg·kg^−1^), Pb (2.50–10.92 mg·kg^−1^), Cu (3.63–10.49 mg·kg^−1^), and Zn (0.02–0.06 mg·kg^−1^, except S3) were highest at S5. Sodium and mercury showed the greatest levels at S3 and S1, respectively, while Se, Ag, and Cd did not vary significantly among sites (Table 2 and Table 3).

### 3.2. Enrofloxacin and Ciprofloxacin

ENR and its secondary metabolite CIP were detected in the aquaculture water bodies and sediment at 6 or 5 sampling points, with a detection rate of 100%. In the water samples, ENR (16.68~3215.95 mg·kg^−1^) and CIP (3.90~459.60 mg·kg^−1^) in S6 were higher than those in other sample points, and the average concentrations were in the following order, S6 > S3 > S4 > S1 > S5 > S2 (Table 4). Within the S6, the order in different seasons was autumn > winter > summer > spring. In the sediment samples, the average concentrations of ENR (41.43~133.67 mg·kg^−1^) were in the following order, S5 > S6 > S3 > S2 > S1, while CIP (12.36~23.71 mg·kg^−1^) were in the following order, S3 > S5 > S1 > S2 > S6, and with the highest levels in spring.

**Table 1:** The water quality of well, river and pond samples from American shad culture companies.

Samples	TN, <6 mg·L^−1^	TP, <0.8 mg·L^−1^	NH_3_-N, <0.2 mg·L^−1^	COD_Mn_, <25 mg·L^−1^	TSS, <100 mg·L^−1^	pH, 6~9
S1 (pond water from the river)	3.58 ± 0.28 b	0.21 ± 0.10 b	**0.92 ± 0.02** c	9.84 ± 1.60 ab	**103.80 ± 23.80** b	7.72 ± 0.30 a
S2 (adult fish pond)	3.88 ± 0.20 b	0.21 ± 0.01 b	**1.52 ± 0.02** b	12.12 ± 2.30 a	**129.67 ± 29.10** ab	7.70 ± 0.30 a
S2 (juvenile fish pond)	2.88 ± 0.10 c	0.22 ± 0.01 b	**1.71 ± 0.01** a	9.60 ± 1.70 b	**196.67 ± 32.40** a	7.82 ± 0.40 a
S3 (well water)	1.22 ± 0.01 d	0.05 ± 0.01 d	**0.22 ± 0.01** f	6.47 ± 1.30 b	**118.00 ± 21.30** b	7.51 ± 0.20 a
S3 (greenhouse pond)	2.93 ± 0.13 c	0.25 ± 0.04 b	**0.35 ± 0.01** e	7.28 ± 1.40 b	**146.00 ± 32.00** ab	8.14 ± 0.10 a
S4 (well water)	3.71 ± 0.14 b	0.30 ± 0.01 b	**0.50 ± 0.01** d	9.81 ± 1.90 ab	**134.00 ± 31.20** ab	7.34 ± 0.40 a
S4 (pond water)	**13.30 ± 2.00** a	**0.80 ± 0.02** a	**0.60 ± 0.02** d	10.00 ± 1.80 ab	**142.00 ± 26.10** ab	8.10 ± 0.10 a
S5 (well water)	2.51 ± 0.11 c	0.11 ± 0.01 c	**0.35 ± 0.01** e	10.76 ± 2.10 ab	**112.67 ± 22.00** b	7.92 ± 0.30 a
S5 (pond water)	4.41 ± 0.29 b	0.19 ± 0.01 b	**1.79 ± 0.01** a	10.13 ± 2.30 ab	**138.00 ± 19.80** ab	8.09 ± 0.20 a

Note: Water samples were collected from the river and the well water. S1, Wuxi Longchao Ecological Agricultural Technology Co., Ltd. (River water), S2, Jiangyin Shengang Sanxian Breeding Co., Ltd., S3, Changzhou Noah Ark Agricultural Technology Co., Ltd. (Well water), S4, Changzhou Tanshi Special Aquaculture Co., Ltd. (Pond water), S5, Yangzhong Jiangzhiyuan Food Industry Co., Ltd. (Pond water), S6, Anhui Changjiangyuge Fishery Co., Ltd. Different lowercase letters showed significant levels, the bolded contents revealed higher than the standards.

**Table 2:** The content of heavy metals in the American shad water (μg·L^−1^) and sediment (mg·kg^−1^) environment.

Medium	Sampling Points	Mn (μg·L^−1^)	Fe (μg·L^−1^)	Co (μg·L^−1^)	Ni (μg·L^−1^) <50 μg·L^−1^	As (μg·L^−1^) <50 μg·L^−1^	Se (μg·L^−1^)	Sr (μg·L^−1^)	Cd (μg·L^−1^), <5 μg·L^−1^	Ba (μg·L^−1^)	Pb (μg·L^−1^) <50 μg·L^−1^	Cu (μg·L^−1^) <10 μg·L^−1^	Zn (mg·L^−1^) <0.1 mg·L^−1^
water	S1	ND d	ND c	0.13 ± 0.03 b	1.84 ± 0.64 b	5.05 ± 1.27 b	0.98 ± 0.07 a	333.46 ± 57.68 c	ND b	10.41 ± 1.00 b	ND d	4.61 ± 1.43 b	0.06 ± 0.00 a
	S2	ND d	ND c	0.06 ± 0.02 c	2.86 ± 0.25 a	1.07 ± 0.13 d	1.00 ± 0.05 a	318.95 ± 70.94 c	ND b	8.16 ± 0.47 b	ND d	1.25 ± 0.15 d	0.07 ± 0.00 a
	S3	0.56 ± 0.08 b	1.07 ± 0.44 a	0.13 ± 0.02 b	2.31 ± 0.26 a	4.87 ± 1.41 b	1.06 ± 0.15 a	562.85 ± 28.63 a	ND b	3.65 ± 0.91 c	5.53 ± 0.35 b	7.77 ± 0.79 a	0.07 ± 0.00 a
	S4	5.00 ± 0.09 a	ND c	0.19 ± 0.11 a	2.44 ± 0.17 a	3.77 ± 1.09 c	1.06 ± 0.13 a	410.08 ± 16.61 b	ND b	17.47 ± 1.09 a	0.27 ± 0.11 c	3.83 ± 0.18 b	0.06 ± 0.00 a
	S5	0.05 ± 0.01 c	0.54 ± 0.12 b	0.11 ± 0.05 b	0.38 ± 0.12 c	9.82 ± 0.09 a	1.38 ± 0.11 a	171.20 ± 7.03 d	ND b	5.06 ± 0.16 c	19.98 ± 1.74 a	2.05 ± 0.28 c	0.07 ± 0.00 a
	S6	ND d	ND c	0.14 ± 0.06 b	0.57 ± 0.04 c	3.47 ± 0.13 c	1.17 ± 0.14 a	455.72 ± 12.04 b	0.05 ± 0.01 a	18.23 ± 2.01 a	0.44 ± 0.02 c	1.30 ± 0.12 d	0.07 ± 0.00 a
		**Mn (mg·kg^−1^)**	**Fe (mg·kg^−1^)**	**Ag (mg·kg^−1^)**	**Ni (mg·kg^−1^)**	**As (mg·kg^−1^)**	**Se (mg·kg^−1^)**	**Cr (mg·kg^−1^)**	**Cd (mg·kg^−1^)**	**Hg (mg·kg^−1^)**	**Pb (mg·kg^−1^)**	**Cu (mg·kg^−1^)**	**Zn (mg·kg^−1^)**
sediment	S1	11.97 ± 1.31 c	525.52 ± 18.21 b	0.01 ± 0.00 a	5.73 ± 0.42 b	1.69 ± 0.13 b	0.20 ± 0.04 a	9.25 ± 1.18 b	0.02 ± 0.01 a	0.13 ± 0.03 a	2.93 ± 0.53 b	3.63 ± 0.79 c	0.02 ± 0.00 b
	S2	12.70 ± 1.83 c	450.47 ± 29.36 b	0.00 ± 0.00 a	5.16 ± 1.06 b	1.13 ± 0.26 b	0.20 ± 0.06 a	7.80 ± 1.75 b	0.02 ± 0.01 a	ND b	2.50 ± 0.73 b	3.82 ± 1.08 c	0.02 ± 0.00 b
	S3	18.49 ± 1.19 b	539.85 ± 49.90 b	0.01 ± 0.00 a	6.36 ± 0.92 b	1.83 ± 0.16 b	0.22 ± 0.03 a	9.61 ± 1.09 b	0.09 ± 0.01 a	ND b	3.05 ± 1.01 b	4.90 ± 0.98 b	0.04 ± 0.02 ab
	S4	-	-	-	-	-	-	-	-	-	-	-	-
	S5	29.94 ± 4.43 a	762.26 ± 52.53 a	0.03 ± 0.00 a	10.18 ± 0.75 a	6.27 ± 0.57 a	0.29 ± 0.02 a	12.72 ± 0.87 a	0.23 ± 0.01 a	ND b	10.92 ± 0.73 a	10.49 ± 0.60 a	0.06 ± 0.00 a
	S6	14.47 ± 3.39 c	500.96 ± 43.43 b	0.00 ± 0.00 a	5.70 ± 0.49 b	1.39 ± 0.12 b	0.17 ± 0.02 a	8.42 ± 0.64 b	0.02 ± 0.01 a	ND b	2.51 ± 0.38 b	3.85 ± 0.72 c	0.02 ± 0.00 b

Note: The different lowercase letters stand for different significant levels. ND stands for no detection. The limit was according to GB11607-1989 of China for heavy metals in water samples.

**Table 3:** The content of metal ions in the American shad water (μg·L^−1^) and sediment (mg·kg^−1^) samples.

Medium	Sampling Points	Li (μg·L^−1^)	Na (μg·L^−1^)	Mg (μg·L^−1^)	Al (μg·L^−1^)	K (μg·L^−1^)	Ca (μg·L^−1^)
water	S1	17.07 ± 4.47 b	14329.97 ± 398.18 a	1550.47 ± 38.77 c	0.45 ± 0.12 b	684.50 ± 18.69 a	5432.18 ± 105.42 b
	S2	8.71 ± 2.10 c	6068.23 ± 188.74 c	2692.39 ± 76.86 b	0.32 ± 0.05 c	273.58 ± 89.73 e	7150.73 ± 179.04 a
	S3	136.51 ± 10.55 a	17753.26 ± 99.33 a	2542.42 ± 67.51 b	0.45 ± 0.11 b	236.39 ± 12.22 e	3994.18 ± 153.03 c
	S4	19.04 ± 4.33 b	9443.96 ± 212.33 b	3740.67 ± 70.59 a	0.44 ± 0.14 b	432.94 ± 34.99 c	6754.77 ± 346.13 a
	S5	18.62 ± 2.52 b	2556.60 ± 37.56 d	1419.67 ± 67.81 c	0.83 ± 0.14 a	397.91 ± 15.73 d	2261.61 ± 57.68 d
	S6	10.80 ± 4.63 c	2989.24 ± 132.74 d	1647.38 ± 72.40 c	0.55 ± 0.14 b	566.81 ± 27.38 b	7016.82 ± 125.21 a
			**Na (mg·kg^−1^)**	**Mg (mg·kg^−1^)**		**K (mg·kg^−1^)**	**Ca (mg·kg^−1^)**
sediment	S1		5.86 ± 0.67 b	88.72 ± 8.22 bc		39.56 ± 4.52 c	170.29 ± 18.27 d
	S2		2.77 ± 0.82 c	65.49 ± 13.51 c		39.62 ± 4.14 c	168.85 ± 11.68 d
	S3		8.31 ± 0.50 a	97.80 ± 15.85 b		54.41 ± 6.97 ab	306.25 ± 20.26 b
	S4		-	-		-	-
	S5		4.22 ± 0.31 b	294.87 ± 15.23 a		69.29 ± 5.84 a	693.51 ± 27.29 a
	S6		2.82 ± 0.26 c	88.04 ± 9.76 bc		47.05 ± 3.66 b	236.55 ± 12.16 c

Note: The different lowercase letters stand for different significant levels. ND stands for no detection. The sediment sample from S4 was not collected because there is a cement pool.

**Table 4:** The content of enrofloxacin (ENR) and ciprofloxacin (CIP) in American shad water and sediment samples (mg·kg^−1^).

Water	ENR Avg.	ENR in Spring	ENR in Summer	ENR In Autumn	ENR in Winter	CIP Avg.	CIP in Spring	CIP in Summer	CIP in Autumn	CIP in Winter
S1	32.37 ± 1.90 d	29.63 ± 1.59 d	19.57 ± 1.07 d	11.2 ± 1.03 d	54.97 ± 6.26 d	5.98 ± 0.85 c	5.47 ± 1.11 c	4.30 ± 0.45 d	2.00 ± 0.12 e	9.50 ± 0.26 d
S2	16.68 ± 1.39 e	13.95 ± 2.47 e	19.20 ± 0.88 d	27.7 ± 0.89 c	8.63 ± 0.40 f	3.81 ± 0.15 d	3.95 ± 0.16 d	4.57 ± 0.19 d	5.10 ± 0.18 d	2.10 ± 0.35 f
S3	1532.10 ± 149.61 b	40.10 ± 1.59 c	744.20 ± 21.54 b	3486.60 ± 43.29 b	1875.5 ± 26.47 b	401.20 ± 31.76 a	6.00 ± 0.51 c	313.30 ± 30.56 b	537.6 ± 60.20 b	747.70 ± 65.32 b
S4	80.33 ± 4.96 c	100.05 ± 10.04 b	96.63 ± 4.78 c	7.23 ± 0.41 e	117.53 ± 17.26 c	29.25 ± 9.14 b	31.31 ± 1.34 b	34.64 ± 1.28 c	15.86 ± 2.67 c	35.31 ± 3.11 c
S5	18.63 ± 1.26 e	18.96 ± 2.34 e	9.56 ± 1.27 e	9.74 ± 1.58 d	36.45 ± 2.41 e	3.90 ± 0.17 d	3.85 ± 0.21 d	2.71 ± 0.13 e	2.75 ± 0.34 e	6.48 ± 0.39 e
S6	3215.95 ± 153.10 a	1552.30 ± 68.92 a	2473.71 ± 150.87 a	6268.39 ± 142.35 a	3239.98 ± 116.23 a	459.60 ± 20.12 a	185.42 ± 10.34 a	386.55 ± 17.04 a	894.83 ± 64.38 a	455.88 ± 49.95 a
**Sediment**	**ENR Avg.**	**ENR in Spring**	**ENR in Summer**	**ENR in Autumn**	**ENR in Winter**	**CIP Avg.**	**CIP in Spring**	**CIP in Summer**	**CIP in Autumn**	**CIP in Winter**
S1	41.43 ± 1.23 c	28.17 ± 2.47 e	48.57 ± 3.67 b	25.52 ± 3.10 d	57.12 ± 4.06 c	21.86 ± 1.74 a	15.12 ± 1.36 b	18.21 ± 2.08 b	30.25 ± 1.39 b	21.78 ± 1.45 a
S2	65.67 ± 3.69 b	47.23 ± 2.56 d	24.36 ± 1.09 c	80.56 ± 4.11 c	74.14 ± 5.21 b	20.56 ± 1.72 a	14.14 ± 2.00 b	35.10 ± 3.14 a	17.02 ± 1.77 cd	21.36 ± 1.05 a
S3	70.14 ± 4.91 b	72.14 ± 5.68 c	124.15 ± 6.23 a	20.15 ± 1.36 d	65.53 ± 2.14 bc	23.71 ± 1.04 a	37.12 ± 2.18 a	20.53 ± 2.74 b	21.52 ± 2.36 c	22.52 ± 1.36 a
S4	-	-	-	-	-	-	-	-	-	-
S5	133.67 ± 6.14 a	227.25 ± 9.22 a	121.36 ± 7.23 a	184.12 ± 10.14 a	74.53 ± 4.99 b	23.67 ± 1.29 a	33.12 ± 1.42 a	15.52 ± 2.18 c	46.23 ± 2.45 a	16.12 ± 1.38 b
S6	125.36 ± 7.44 a	98.26 ± 4.21 b	120.36 ± 4.77 a	130.69 ± 7.78 b	94.78 ± 8.41 a	12.36 ± 1.34 b	9.58 ± 0.75 c	10.33 ± 1.23 d	14.77 ± 1.06 d	13.05 ± 1.06 c

Note: The average concentrations of ENR and CIP analyzed per month for one year, and their concentrations for each season have also been calculated. Spring, summer, autumn, and winter were March~May, June~August, September~November, December~February. The different lowercase letters stand for different significant levels.

### 3.3. ENR Degradation Bacteria and Their Optimization Conditions

**Figure 2:**
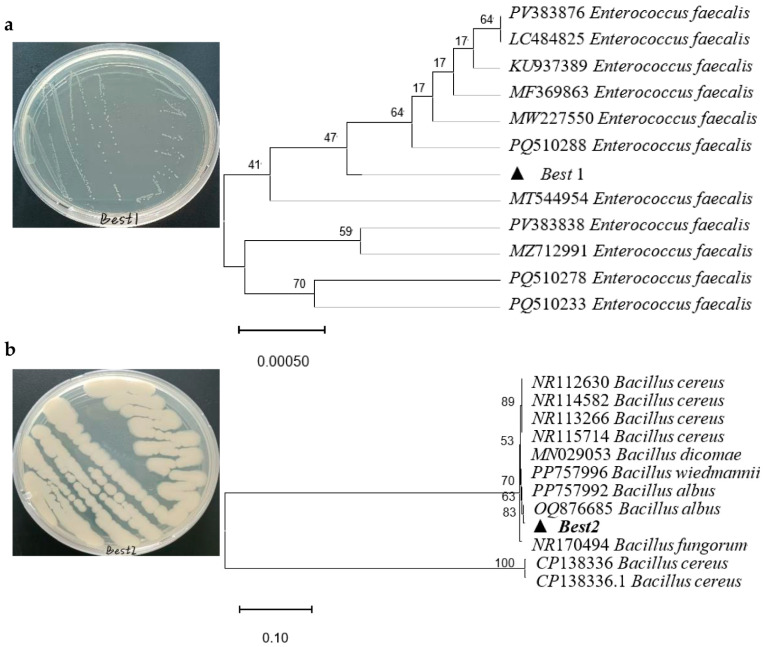
The phylogenetic trees of Best1 (**a**) and Best2 (**b**). (**a**) gene licence data for *Enterococcus faecalis* were PV383876, LC484825, KU937389, MF369863, MW227550, PQ510288, MT544954, PV383838, MZ712991, PQ510278, PQ510233. (**b**) gene licence data for *Bacillus* were NR112630, NR114582, NR113266, NR115714, MN029053, PP757996, PP757992, OQ876685, NR170494, CP138336, CP138336.1. The black triangle in the figure showed the isolated bacterias in this study.

**Figure 3:**
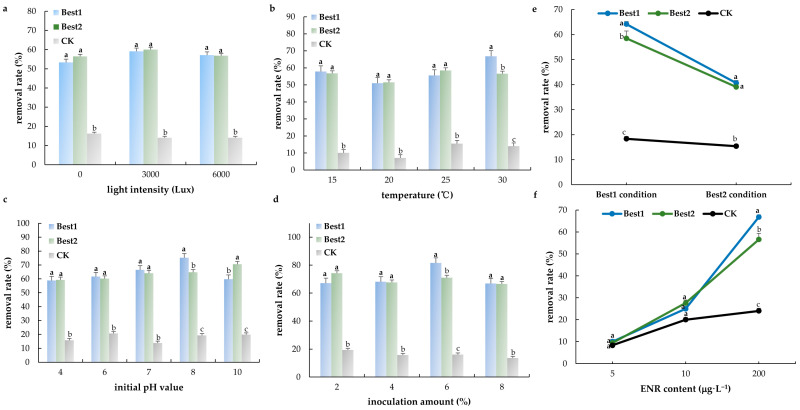
The removal rate of ENR for different affecting factors (*n* = 3), like light intensity (**a**), temperature (**b**), initial pH (**c**), and inoculation amount (**d**). (**e**) the removal rate of ENR for Best1 and Best2 in its optimization conditions, (**f**) the removal rate of bacteria verification for 5~200 μg·L^−1^ ENR. Different lowercase letters showed significant levels.

Two highly efficient degradation strains were obtained through screening and named Best1 and Best2, respectively. After DNA extraction and PCR amplification of these two strains, two amplification product bands were obtained. The sequencing results of the amplified products were submitted to the NCBI database for analysis. Homology comparison was performed using BLAST, and several 16S rDNA gene sequences showing high similarity (>95%) to Best1 and Best2 were selected. After multiple sequence alignment, the findings showed that Best1 was closely related to *Enterococcus faecalis* (Figure 2a), while Best2 shared high homology with *Bacillus subtilis* (Figure 2b). With the increase in light intensity, the degradation rates of enrofloxacin by Best1 and Best2 showed a trend of first increasing and then decreasing. The degradation rates reached their highest at a light intensity of 3000 Lux (Figure 3a), which were 59.10 ± 0.50% and 60.05 ± 2.15%, respectively. The rate of decrease in shaking speed in the blank group was 14.10 ± 0.54%. The degradation rate of Best1 shows a trend of first decreasing and then increasing with increasing temperature, reaching its highest value (66.80 ± 3.30%, Figure 3b) at 30 °C, while the degradation rate of the blank group at this temperature is 14.00 ± 1.60%. The degradation rate of Best2 shows a fluctuating trend with temperature, reaching its peak at 25 °C (58.50 ± 2.00%), while the degradation rate of the blank group at this temperature is 15.50 ± 2.84%.

The degradation rate of the Best1 strain showed a trend of first increasing and then decreasing with increasing pH, reaching the highest degradation rate (75.25 ± 1.05%, Figure 3c) at pH 8.0. The degradation rate of the blank control group under this pH condition was 19.30 ± 1.10%. The degradation rate of the Best2 strain showed a continuous and slow upward trend with increasing pH, reaching the highest degradation rate (70.55 ± 0.85%) at pH 10. The degradation rate of the blank control group under this pH condition was 19.80 ± 0.30%. Both strains (Best1 and Best2) showed a degradation trend of first increasing and then decreasing, with the peak degradation rate occurring under the condition of 6% inoculation volume. The degradation rate of Best1 reached 81.60 ± 2.10% (Figure 3d) at a 6% inoculation dose, while the degradation rate of Best2 decreased by 71.00 ± 3.00% at the same inoculation dose. The degradation rate of the blank control group under this condition was 16.10 ± 2.30%.

The optimal combination of factors for Best1 is a temperature of 30 °C and a pH of 8; The optimal combination of factors for Best2 is a temperature of 25 °C and a pH of 10. Best1 and Best2 had the highest degradation rates under their respective optimal factor combinations, which were 64.20 ± 0.80% and 58.50 ± 1.73%, respectively. The degradation rate of the blank group was 18.35 ± 2.02% (Figure 3e). The different concentrations of ENR (5~200 μg·L^−1^) were used for verification, and the results showed the order of removal rate in this degradation bacteria was in the following order: Best1 > Best2 > CK (Figure 3f).

**Figure 4:**
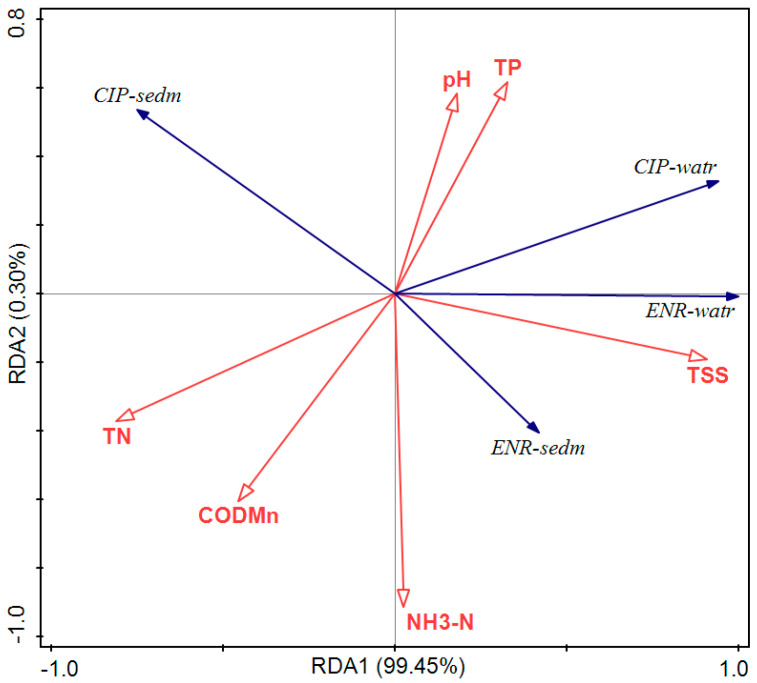
RDA analyses for water quality, ENR and CIP concentrations. “watr” and “sedm” stand for water and sediment samples.

ENR and CIP concentrations in the water samples had a positive correlation with pH (Figure 4), TP, and TSS; however, they had a negative correlation with TN, COD_Mn_. ENR and CIP concentrations in the sediment samples had a positive and negative correlation with TSS.

## 4. Discussion

### 4.1. Environmental Stressors and the Reliance on Antibiotics in American Shad Culture

American shad, an important economic anadromous fish with a life history of transition from marine to diadromous adapting to changes in salinity, has emerged as a promising species for recirculating aquaculture systems (RAS) in China [10,31]. Genomic and transcriptomic work has revealed interesting physiological traits, particularly in lipid metabolism, cytoskeletal remodelling, and osmosensory signalling during migration between saline and freshwater habitats [32,33,34]. Genes involved in fatty acid synthesis and degradation appear to be under strong selective pressure, which may explain some of the differences in lipid physiology across clupeiform fishes [34]. Climate change, like seasonal shifts, extreme events, adds another layer of complexity by altering thermal regimes, like long-term warming, and habitat use [35], with downstream effects on circadian rhythm, nutrient processing, and stress regulation [36,37,38,39].

Elevated temperatures increase metabolic demand and physiological stress in shad, though these effects can be partially offset by dietary supplements such as probiotics. For instance, *Lactococcus lactis* has been shown to stimulate fatty acid β-oxidation, reduce oxidative stress, and lower cortisol secretion [40,41]. Similarly, algae provide a natural carbon source and support microbial stability in culture systems [42]. At the same time, pond hydrodynamics—whether water is flowing or stagnant—play a decisive role in shaping antibiotic distribution and removal efficiency [9].

Within this context, the widespread detection of enrofloxacin (ENR) and its metabolite ciprofloxacin (CIP) in both water and sediment is concerning. ENR is known to disturb lipid metabolism, provoke oxidative damage, and trigger inflammatory pathways in fish [10,11,18]. Because of its strong affinity for sediments with high organic content [43], ENR tends to accumulate in pond bottoms, particularly in cement-lined systems where microbial diversity is low and degradation capacity is poor. These factors explain the persistence of ENR residues across seasons and highlight the need for targeted remediation strategies [44].

### 4.2. Factors Shaping ENR Degradation and Optimization of Microbial Remediation

Biological degradation of ENR, whether by bacteria or algae, is increasingly viewed as a practical and sustainable approach for aquaculture water treatment [45]. Several bacterial groups—including *Lactococcus*, *Bacillus*, and *Enterobacter*—have shown promising degradation capacity in freshwater environments [23]. In marine systems, members of *Rhodobacteraceae* and *Alcanivoracaceae* are more commonly implicated [46]. Certain fungi, such as *Cladosporium*, *Talaromyces*, and *Bjerkandera*, can also degrade ENR efficiently, often reaching removal rates above 90% under optimized conditions [47,48,49,50]. Even greater success has been reported when bacteria and algae are combined, as in *Chlorella*-based consortia, where enzymatic activity, biosorption, and photosynthetically driven oxidation act in concert to accelerate removal, like using eukaryotic-bacterial symbiotic membrane less bio–electrochemical system [51], microalgae via shifts in microbial communities [52], and microalgal-bacterial granular sludge [53].

Environmental variables are central to the performance of these systems. Light intensity [54], temperature [47,55,56,57,58,59], pH [54], salinity [58], and inoculum size [55] all affect degradation efficiency. Although thermal hydrolysis at 160–170 °C can remove more than 80% of ENR, such harsh conditions are impractical for aquaculture [60,61]. More modest strategies—for example, cultivating *Bacillus* species at 25–30 °C, pH 8–10, with 6% inoculum under moderate light—can achieve over 60% removal within three days, which makes them realistic candidates for farm-level interventions.

One of the more difficult challenges is the persistence of ENR in sediments. The elimination half-life for drug in fish can exceed 20 h [21], which contributes to chronic liver, intestinal, and oxidative stress injuries [10,11]. To address this, integrated remediation approaches are gaining attention, including bacterial–algal consortia and biochar-based carriers that can stabilize microbes while enhancing adsorption capacity [62]. These strategies offer a way forward for tackling residues embedded in sediments, not just in the water column. The hydrodynamics from ponds in the diverging area become complicated because of the basin hydrological conditions, making the distribution of antibiotics largely uncertain. ENR concentrations are mainly affected by sediment particle size and oxygen content. The water physical and chemical properties, altered by the river basin hydrological conditions, play an important role in influencing the distribution of ENR concentrations [63].

### 4.3. Toward Sustainable Aquaculture and One Health

Antibiotics in aquaculture rarely occur in isolation, and ENR residues in fishes can pose a risk to human health [64]. They co-exist with heavy metals, organic pollutants, and sometimes microplastics, creating a cocktail of stressors that compromise fish health and increase risks to consumers through trophic transfer [65,66,67,68,69,70]. The current study showed water quality had a significant relationship with the concentrations of ENR and CIP in the water and sediment samples, especially TN and TSS had a negative relationship with water and sediment CIP. Our observations, together with previous studies [71,72], support the idea that microbial and algal preparations improve not only water quality but also microbial diversity, thereby buffering fish against antibiotic stress. Beyond microbes, natural phytochemicals such as resveratrol from *Polygonum cuspidatum* offer an additional layer of protection, acting both as hepatoprotectants and as potential phytoremediation agents [73,74,75,76,77]. CIP addition affected the concentrations of TSS [78], while higher TSS concentrations enhanced the removal rate of CIP through sorption [79]. Activated carbon or biochar adsorption is a promising technique for antibiotics in the field of wastewater treatment [80]. Another study showed CIP exposure suppressed denitrification and phosphorus uptake processes through decreasing the activities of nitrite reductase and polyphosphate kinase [81].

Several limitations remain. We were not able to determine fully optimized conditions for bacterial degradation using orthogonal design, and the precise pathways of ENR catabolism remain unresolved. Future work should therefore combine high-resolution metabolomics with microbial genomics to trace degradation intermediates, evaluate the toxicity of breakdown products, and refine integrative systems that bring together microbes, algae, and biochar for scalable applications, like antibiotic removal technology in the field of pond water restoration.

Taken together, these findings point toward a sustainable pathway for aquaculture management. By linking molecular insights with ecological engineering, it is possible to reduce antibiotic contamination, safeguard the welfare of cultured species such as American shad, and mitigate risks to both environmental and human health.

## 5. Conclusions

American shad (*Alosa sapidissima*) is now one of the most valuable farmed fish in China, but its culture is increasingly threatened by water quality problems, heavy metal contamination, and the widespread use of antibiotics. Our year-long monitoring of six farming sites showed that ammonia nitrogen and suspended solids often exceeded safety standards, while toxic metals such as copper, cadmium, arsenic, and lead were detected at concerning levels in both water and sediments.

Even more striking was the consistent presence of enrofloxacin (ENR) and its metabolite ciprofloxacin (CIP), which were found in every water and sediment sample. Concentrations reached several thousand milligrams per kilogram in some sites, raising serious questions about their frequent use in shad culture. Because these drugs can accumulate in fish tissues, prolonged exposure may compromise fish health, particularly liver function, and pose risks to consumers.

On a more encouraging note, we isolated two bacterial strains, *Enterococcus* and *Bacillus*, that were able to degrade ENR under optimized conditions. While these results point to the potential for bioremediation, practical solutions will likely require more integrated strategies. Combining bacteria with algae or engineered biochar could provide effective, low-cost approaches to reducing antibiotic residues in aquaculture systems.

In short, ensuring the sustainability and safety of American shad farming will depend on both controlling pollution inputs and advancing bioremediation technologies that can protect fish health and reduce risks to consumers.

## Data Availability

The original contributions presented in this study are included in the article. Further inquiries can be directed to the corresponding author(s).
